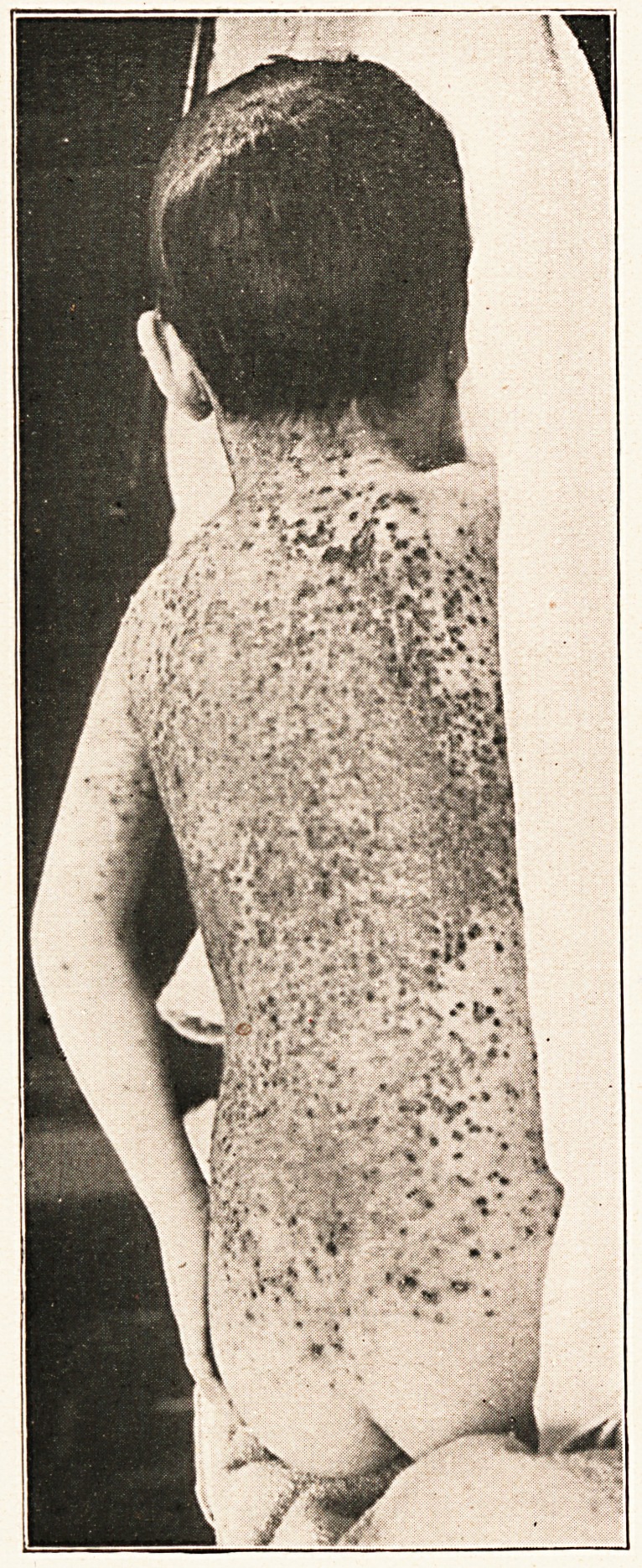# A Case of Anomalous Cutaneous Eruption Showing a Bacillus Morphologically Corresponding with the Klebs-Lœffler, and Clearing up under Diphtheritic Antitoxin

**Published:** 1910-09

**Authors:** W. Kenneth Wills

**Affiliations:** Physician, Skin Department, Bristol General Hospital.


### A CASE OF ANOMALOUS CUTANEOUS ERUPTION SHOWING A BACILLUS MORPHOLOGICALLY CORRESPONDING WITH THE KLEBS-LŒFFLER, AND CLEARING UP UNDER DIPHTHERITIC ANTITOXIN.

BY

W. Kenneth Wills, M.A., M.B., B.C. (Cantab.),

*Physician, Skin Department, Bristol General Hospital.*

---

The part the diphtheria-carrier plays in the production of epidemics has only lately been investigated, and it has been found that the infective agent has remained almost indefinitely in the nasal passages, or in otorrhœal discharges, in spite of all endeavours to disinfect. Upon these chronic infections the use of antitoxin seems to have little or no effect. On the other hand, immunity to the diphtheritic toxin seems to be conferred upon the carrier himself, his own yield of antitoxin being no doubt sufficient to counteract the toxin absorbed from the nasal passages, etc.

That the nasal passages or the ear should form such a nidus does not seem wonderful, considering the impossibility, apart from extensive operative procedure, of reaching the various cells and sinuses which harbour the infection. That the skin should become infected by the Klebs-Lœffler bacillus is readily understood, and it is a matter of fairly common experience to find abrasions and cuts with false membranes during an attack of diphtheria, or even affording the portal of entry of the infection. These infections, however, partake of the nature of an acute infection, they are followed by toxic symptoms, and are amenable to local disinfection. It has only lately come to our knowledge that cutaneous eruptions may harbour, or perhaps even be caused by, the Klebs-Lœffler bacillus.

Slater[1] quotes such a case where the bacillus of diphtheria

[1] *Lancet*, 1908, i, 15.

in a strain the toxicity of which was proved by its lethal effect upon a guinea-pig in ten days, was recovered from a vesicular, cutaneous eruption. No antiseptic used was sufficient to disinfect the lesions, although they were so superficial as to heal eventually without scarring. Altogether the patient had had the cutaneous eruption for three years without constitutional symptoms, and it was only when diphtheritic antitoxin was administered that any appreciable improvement occurred. This improvement, which set in after the first dose of 2,000 units of antitoxin, was only temporary, and seven subsequent doses were required before the tendency for the rash to recrudesce was arrested, and before growth failed to occur in inoculated tubes. The spleen was not enlarged, the heart was normal, and there was no albuminuria.

Eddowes[1] quotes a similar case in a girl æt. 11, who presented severe ecthymatous bullæ on the arms, hands and lips. There were traces of a ruptured vesicle in the anterior pillar of the fauces, but no false membrane. Klebs-Lœffler bacilli were obtained from the lesions, and proved lethal to a guinea-pig in twenty-seven hours. This patient recovered without antitoxin.

The case which we wish to present is unfortunately imperfect in one important respect, viz. the toxicity test, but yet, notwithstanding, it seems to be of sufficient interest to warrant publication, if only for the purpose of drawing further attention to the part that diphtheria may play in maintaining cutaneous eruptions over protracted periods, without the specific toxic effects expected with diphtheria. One very important point, if no other, is established by this case, viz. that although the absorption of toxin must be continuous throughout the disease and the consequent manufacture of sufficient antitoxin to prevent specific toxic symptoms, the use of antitoxin, given hypodermically, was attended by instant improvement in the cutaneous eruption.

The following notes have been kindly communicated by Dr. Elliott Glenny, who was originally in charge of the case :—

[1] *Lancet*, 1908, i, 282.

*J. P., æt. 6, showing profuse pustulo-vesicular rash from which was recovered a bacillus morphologically indistinguishable from the Klebs-Læffler B. diphtheriæ, and which cleared up after the use of diphtheritic antitoxin.*

(*Photo. by* Dr. FLETCHER.)

J.P., 6 years old, was brought under observation for enlarged glands in the neck on December 16th, 1909.

His previous history shows that he had, six months ago, " an abscess which discharged through the right nostril." Since then glands have slowly increased in size on the left side of the neck without obviously affecting the general health.

On December 16th a slight follicular tonsillitis was observed on the left side. This was not attended with pyrexia or alteration of the general condition, and the patient " enjoyed his games and sang with the other children." The throat was swabbed twice daily with 1 in 60 carbolic acid, and potassium chlorate and ferric chloride were given internally.

The glands gradually increased during the next six weeks, and fluctuated, threatening once or twice to break down. The left tonsil slowly changed its appearance from a follicular tonsillitis to a punched-out ulcer.

At the beginning of February the throat began to be painful, and tablets of menthaform were substituted for the carbolic swabbing.

In the middle of February the skin over the shoulders became *scaly*, and this condition spread in a couple of weeks to the whole trunk, and to a lesser extent to the thighs, arms, neck and face, but not involving the hands, feet or scalp. By the end of the month, while the scaly condition was extending, the parts first involved were showing more inflammatory changes, and a small amount of serous discharge appeared, and the scales were shed, leaving a raw surface. At the end of the first week of March the whole of the trunk had become " raw," the conjunctiva had become involved, the gums and lips were also ulcerated, and the whole condition of the child most pitiable. The discharge had soon become purulent and offensive, but no local treatment seemed in any way to allay the symptoms. The temperature was of a septic type, but did not run very high.

It was at this time that I was asked to see the case, and I found him presenting the appearance of a profound toxæmia. His face was pallid, with sores round the lips, which were swollen. He had a dressing over the glands behind the left ear on account of suppuration. He seemed listless and apathetic, but would answer questions. Occupying the back, chest and abdomen for the most part, with a few scattered lesions on the face and limbs, was a copious vesiculo-pustular rash. On the margins of the efflorescence the lesions were markedly discrete and of a conical shape with a depressed centre. This was due to their being formed round the pilo-sebaceous follicles. Most of the lesions were pustular, though some were papular, while in the centre of the affected areas were crusts and erosions. The intervening skin was in parts darkly erythematous, but round the more recent pustules there was only a narrow erythematous ring. In some there were evidences of hemorrhage.

Inside the mouth there were one or two shallow ulcers, situated on the lips and soft palate. One of these had a greyish pellicle, which suggested to my mind that there might be a diphtheritic infection. There was a central streak of hemorrhage on the uvula. There was conjunctivitis of both eyes, the left being the worse, but the exudate was not characteristic of diphtheria.

Cultures were made from the throat lesions, from the nose, eye, and the skin lesions, and all were returned "positive Klebs-Lœffler." The patient was accordingly admitted into Ham Green Fever Hospital.

*March 8th*, 1910.—On admission Dr. Fletcher, to whom I am indebted for these notes, injected 10,000 units of antitoxin, and upon the bacteriological report accompanying the case he investigated the bacteriology, and found :—

| | |
|---|---|
| NOSE (Toluidine) .. | Fairly long, bipolar-stained bacilli, some only unipolar. |
| ,, (Lœffler) .. .. | Long and short granular bacilli, diplococci. |
| EYE (Toluidine) .. | Shorter granular bacilli, bipolar and unipolar. |
| ,, (Lœffler) .. .. | Long granular bipolar bacilli, diplo- and ordinary cocci. |
| THROAT (Toluidine) .. | Long and short granular bacilli, and diplococci. |
| ,, (Lœffler) .. | do. do. |
| SKIN (Lœffler) .. .. | Granular bacilli and cocci. |

Almost immediately improvement began to set in after the antitoxin. The conjunctivæ were less infected and less discharge from skin.

March 11th.—2,000 units antitoxin injected. Temperature 100° to 102°.

,, 13th.—Inflammatory reaction of eruption much less, and clearer. Still a few bleeding points on back. Upper lip shows less swelling, and mucous membrane healing. Antitoxin 2,000 units. Temperature normal.

,, 15th.—Antitoxin 2,000 units.

,, 17th.—Eruption healing rapidly, and much cleaner. No exudation or bleeding points. Temperature remains about 99°.

,, 22nd.—Antitoxin 2,000 units. Temperature has risen to 102°.

,, 23rd.—Antitoxin, rash on legs. Faint mitral systolic murmur. Interval, and second sound rather shortened.

,, 28th.—Distinct croupy cough. Colour not so good. Eruption on head worse. Antitoxin 2,000 units.

March 30th.—Harsh, mitral systolic murmur. *Lungs:* Right lung bronchitic, tubular breathing at apex, no dulness. Temperature rising to 104°.

After this time no antitoxin was given, and the patient has shown signs of consolidation of apices of lungs. There was a slight recrudescence of the eruption at the root of the neck and lower part of back on April 2nd, which has improved without further doses of antitoxin.

I saw the boy on March 26th, and was astonished at the improvement not only in the general condition, but in that of the skin. All the pustules on back and front had disappeared, leaving only deeply-pigmented, discrete maculæ. There was some impetiginous rash on the head, of a different nature, in my opinion, from that on the body. This was still persisting slightly.

The local treatment had comprised boric ointment and zinc and mercury ointment applied on lint.

While well aware that this report will be open to the criticism that there is no proof that the Klebs-Lœffler was the causal agent of the cutaneous trouble, I am yet of opinion that the rapid relief of the cutaneous condition by means of the antitoxin injections is of sufficient interest to warrant its publication, and am also of the impression that the cutaneous rash was at least maintained by, if not produced by, the same cause as the mucous membrane ulcerations, all of which have cleared up.

I have to thank Drs. Glenny and Fletcher for their kindness in allowing me to make free use of their notes. Also I am indebted to Dr. Fletcher for the accompanying photographs, taken on March 14th.

## Figures and Tables

**Figure f1:**
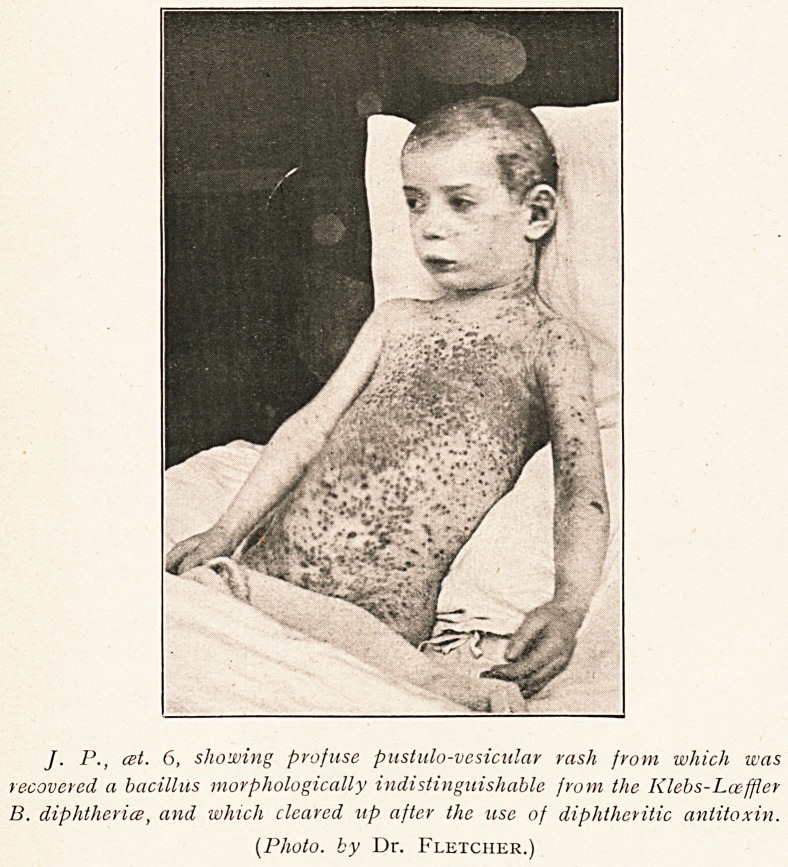


**Figure f2:**